# Editorial Opinion: Value Within the Prosthetic and Orthotic Provision Process

**DOI:** 10.33137/cpoj.v5i1.38442

**Published:** 2022-07-17

**Authors:** S.U. Raschke

**Affiliations:** British Columbia Institute of Technology (BCIT), 3700 Willingdon Avenue, Burnaby, British Columbia, Canada.

**Keywords:** Orthosis, Prosthesis, Health Economics, Prosthetics, Orthotics, Funding, Outcome Measures

## Abstract

This Editorial presents an overview of the uptake of clinical outcome measures in the prosthetics and orthotics sector and considers how the use of objective measures contribute to demonstrating value provided. A decade ago, payors began to demand objective data to document costs vs. benefits from prosthetic and orthotic providers. The speed with which the sector responded to help develop measures and to begin to integrate them into practice is remarkable. This suggests an encouraging resilience and ability to adapt on the part of the sector as other trends such as Values-Based Health Care emerge to challenge the sector.

The recent Special Edition of the Canadian Prosthetics and Orthotics Journal focused on the theme of Health Economics in Prosthetics and Orthotics, highlighting some of the complexities associated with providing optimal solutions to the end-users of prosthetic and orthotic devices. Many people enter the field of prosthetics and orthotics with the desire to connect with and help people, but the underlying reality is that this desire will always be fiscally constrained. For his reason, it is essential to be able to critically analyse one’s work from a cost: benefit perspective and to be able to define and communicate the value of that work to payors whether one works on the clinical or the engineering side of the sector.

An underlying theme in the Special Edition was the significant changes in practices that had emerged once payors began linking reimbursement to the presentation of objective outcome measures a decade ago. At that time prosthetists and orthotists were increasingly required to present objective criteria to communicate and to justify their component and device choices if they wished to be reimbursed. This was a significant shift in the practice paradigm, as previously measurement activities had been limited to device production and fitting related tasks.

The response to this new requirement was a palette of outcome measures developed and validated by prosthetic and orthotic researchers for use in a clinical setting. These measures allowed the quantification and tracking of typical rehabilitation outcomes such as mobility, function, activity levels and pain, along with dimensions intended to capture client satisfaction levels,^1^ allowing prosthetists and orthotists to meet payor requirements. These measures also gave prosthetists and orthotists fluency in the objective language of the medical and scientific communities, allowing them to begin to articulate the previously undocumented interactions with their clients that go beyond the “*simple*” provision of a device and bring “*added value*” to the provision process. The adoption of an objectively anchored clinical practice model helped make visible what had previously been invisible.

At the same time that payors were compelling clinically facing providers to justify their provision decisions, a related pressure was building upstream, at the technical develop-ment level, where payors began to query the costs of new technology solutions. Here the critical question was: What significant, measurable value does more complex, and typically more expensive, technology bring?

The classic approach to answering this question, much as a new pharmacological intervention would be evaluated, is an Evidence Based Medicine (EBM) approach which became the gold standard in the 1990’s. EBM relies on Random Clinical Trials (RCTs) to generate large data sets from which criteria such as Minimum Clinically Important Differences (MCID) and Dosing can be generated to guide policy and funding decisions. Unfortunately, it is not possible to carry out classic RCTs in the prosthetics and orthotics sector due to small patient populations, the high costs of such studies and the technical challenges of setting up double blinded study designs, which are the gold standard for EBM models.

Instead, prosthetics and orthotics focused engineers and researchers turned to Small-N research designs^2^ (e.g. Multiple Baseline, Cross-Over, Repeated Measures, Single-Blinded, etc.) to compare classes of componentry, (i.e. microprocessor vs hydraulic controlled knees or energy storing vs no energy storing prosthetic feet) and results indicated that class of component chosen can lead to measurable differences along a range of dimensions.^3,4^

Through this concentrated, two-pronged research effort spanning clinical aspects and engineering technology, the prosthetics and orthotics sector has been able to transition from an unscientific, artisan-based practice to adopting objective, data-based ways of thinking in a period of ten short years. This is quite an accomplishment. Both clinically focused and technology evaluation research studies continue to be done and there is a growing knowledge base indicating that prosthetic and orthotic technology and interventions create measurable changes and experiences for the users of the devices.

As health care continues to be rationalized, the next emerging stage in health care policy and decision making is trending toward a Values Based Health Care (VBHC) model, which seeks to link funding to outcomes.^5^ This is based on emerging belief in the business and policy press that the prevalent pathways for funding health care based on fee for service, device or process do not necessarily lead to the best or most efficient health care outcomes.^6,7^

VBHC advocates for decision making processes negotiates a compromise between balancing genomic-biomedical-biomechanical aspects of an individual alongside psychosocial and behavioural characteristics, with some consideration of what outcomes matter most to the individual. The model embodies lofty ideals and will be challenging to implement. Determining what treatment goals and objectives are optimal for any given situation will require negotiation and sensitivity as “*optimal*” or “*desired*” as defined by one stakeholder vs another will often be in conflict with each other.^8^ Achieving this balance between stakeholders, including individual patients, will take considerable time and effort.

Further complicating this shift is the lack of transparency and consistency from payors as to what measures and evidence are acceptable and deemed adequate for the existing reimbursement models, creating high levels of confusion and frustration with the processes. VBHC will allow for a much denser data sets to be collected along an expanded number of domains. How this data is to be organized, evaluated and weighted in a timely and fair manner is one of the critical questions that must be answered and those answers must be clear. In reimbursement processes already burdened by a lack of clarity, it will be necessary for payors to work with the sector to establish clear and stable goalposts in the various domains in which data will be collected, if this approach is to be successful.

Were does this new trend leave the prosthetics and orthotics sector? Having navigated the past decade amazingly well, my sense is that the sector is in good shape, but with one critical caveat.

A decade ago, payors began to require objective outcome measures at a time where few such measure existed and were never used in reporting, challenged the prosthetics and orthotics sector to objectively communicate the value they bring. This externally applied pressure led to unprecedented collaboration between clinicians, researchers and professional organizations who rose to the challenge collectively to create astonishing changes in opinions, attitude and practices. The result is the creation of a value focused lens that has become baked into the sector.

The transition has been stressful and disruptive because it was driven by outside forces as opposed to being internally motivated. Considering at where the sector was a decade ago, the transformation is astounding and offers hope. I see a maturity and confidence in the sector that was not present even 15 short years ago. I believe this comes in large part because, for the first time in the history, the sector has had to take a hard, critical look at what they do, why they do it and what value they create and found, perhaps to the surprise of some, that value could be objectively demonstrated. This process allowed the sector to deconstruct the myth of the “*magic in the hands*”^9^ of the clinician and replaced it with a genuine and professional identity, expressed in the common language of science.

The caveat is that, the cascade of next generation health care technology that is now entering the market will create additional, exponential pressures on the sector. Keeping up with, integrating and mastering this technology will require an ongoing, energetic response. There no time for the prosthetics and orthotics sector to sit back, catch its breath and enjoy the past decade’s successes. Instead, effort needs to be scaled up further with respect to building knowledge and expertise in measuring and communicating value.

The future is daunting, but looking back at what has been achieved over the past decade, I believe the sector has successfully created an objectively anchored foundation which will serve it well when navigating what will continue to be uncertain waters.

## DECLARATION OF CONFLICTING INTERESTS

I have no conflicts to interest to declare.

## SOURCES OF SUPPORT

None.
